# Efficacy of Laparoscopic Hepatectomy *versus* Open Surgery for Hepatocellular Carcinoma With Cirrhosis: A Meta-analysis of Case-Matched Studies

**DOI:** 10.3389/fonc.2021.652272

**Published:** 2021-05-07

**Authors:** Yu Pan, Shunjie Xia, Jiaqin Cai, Ke Chen, Xiujun Cai

**Affiliations:** ^1^ Department of General Surgery, Sir Run Run Shaw Hospital, School of Medicine, Zhejiang University, Zhejiang, China; ^2^ Key Laboratory of Endoscopic Technique Research of Zhejiang Province, Hangzhou, China; ^3^ Department of Plastic Surgery, Sir Run Run Shaw Hospital, School of Medicine, Zhejiang University, Zhejiang, China

**Keywords:** laparoscopic hepatectomy, hepatocellular carcinoma, cirrhosis, prognosis, meta-analysis

## Abstract

**Background:**

The role of laparoscopic hepatectomy (LH) in hepatocellular carcinoma (HCC) with cirrhosis remains controversial and needs to be further assessed. The present meta-analysis aimed to compare the surgical and oncological outcomes of LH with those of open hepatectomy (OH) for HCC with cirrhosis.

**Methods:**

The PubMed, Embase, and Cochrane Library databases were searched for studies comparing LH and OH until Mar 2021. Weighted mean differences (WMDs), odds ratios (ORs), and hazard ratios (HRs) were calculated for continuous, dichotomous, and long-term variables, respectively, with 95% confidence intervals (CIs). Subgroup analysis was performed according to different resection types: major resection and minor resection. The meta-analysis was performed using the STATA 12.0.

**Results:**

A total of 16 case-matched studies (784 patients in the LH group and 1,191 patients in the OH group.) were included in this meta-analysis. In terms of primary outcomes, LH was associated with decreased overall complication rate (OR 0.57; 95% CI 0.46 to 0.71; P <0.01), major complication rate (OR 0.52; 95% CI 0.33 to 0.82; P < 0.01), postoperative mortality (OR 0.27; 95% CI 0.11 to 0.66; P  <0.01), 1-y overall survival (OS) rate (HR 0.48; 95% CI 0.31 to 0.73; P <0.01), 2-y OS (HR 0.61; 95% CI 0.45 to 0.83; P < 0.01), and 5-y OS (0.67; 95% CI 0.53 to 0.85; P < 0.01). With respect to secondary outcomes, blood loss (WMD −69.16; 95% CI −101.72 to −36.61; P < 0.01), length of hospitalization (LOH) (WMD −2.65; 95% CI −3.41 to −1.89; P < 0.01), minor complication rate (OR 0.70; 95% CI 0.53 to 0.94; P = 0.02), postoperative liver failure (OR 0.60; 95% CI 0.38 to 0.95; P = 0.03), and postoperative ascites (OR 0.44; 95% CI 0.28 to 0.72; P < 0.01) was lower in LH than in OH. No significant differences in operation time (P = 0.07), transfusion rate (P = 0.05), 1-, 2-, and 5-year DFS rate (1-year, P = 0.08; 2-year, P = 0.08; 5-year, P = 0.23) were noted between LH and OH. Subgroup analysis based on minor resection revealed that LH had similar favored outcomes in comparison with those in the overall pooled analysis. However, LH had a longer operation time than OH in the setting of major resection (P < 0.01).

**Conclusion:**

LH is technically feasible and safe for selected HCC patients with cirrhosis. LH can achieve favored short-term and long-term oncological outcomes in minor liver resection. Laparoscopic major hepatectomy (LMH) seems to offer some advantages over the open approach; however concerns about surgical and oncological safety remain. More evidence on LMH is warranted before expanding its indication to patients with cirrhosis.

## Introduction

Hepatocellular carcinoma (HCC) is the most common primary cancer of the liver and one of the leading causes of cancer-related deaths worldwide (1, 2). Hepatectomy is the commonly used curative treatment strategy for very early- and early-stage HCC patients with preserved liver function. In the early 1990s, with the inception of laparoscopic techniques, initial reports on laparoscopic hepatectomy (LH) were published (3, 4). Since then, the laparoscopic approach has been increasingly accepted in the field of liver surgery. Laparoscopic techniques have been shown to expedite recovery, improve postoperative pain, and result in better cosmesis than the open approach. In the statement of the First International Consensus Conference for Laparoscopic Liver Resection, laparoscopic left lateral segmentectomy was identified as the gold standard approach (5). In 2014, the Second International Consensus Conference for Laparoscopic Liver Resection recommended laparoscopic minor hepatectomy as the standard surgical practice (6).

Most patients with HCC commonly have chronic hepatitis and cirrhosis making liver resection technically demanding. Liver resection is a challenging procedure in the setting of cirrhosis owing to elevated portal pressure and impaired coagulation function in patients with this condition. A retrospective study by Neeff et al. reported that the severity of cirrhosis was correlated with perioperative mortality after hepatectomy (7). The development of devices and techniques for hemostasis has allowed bleeding control in LH. Several efforts have been made to promote the adoption of LH in the treatment of HCC with cirrhosis (8–11). Given the advantages of laparoscopic surgery in terms of minimal invasiveness, LH is expected to be more beneficial for HCC patients with cirrhosis. Several meta-analyses have reported that patients with cirrhosis undergoing LH experienced less blood loss, fewer postoperative complications, and shorter hospital stays than those undergoing open resection (12, 13). Most studies included in these meta-analyses were retrospective and limited to laparoscopic minor resection. Since then, one randomized clinical trial (RCT) and several case-matched studies focusing on HCC with cirrhosis have reported the favored surgical outcomes of LH (14–16). Furthermore, major liver resection is an important curative modality for HCC. Recently, laparoscopic major hepatectomy (LMH) for selected patients with cirrhosis has been reported by several experienced surgeons in a few medical centers (17, 18). Hence, in this study, we aimed to compare the surgical and oncological outcomes of LH with those of open hepatectomy (OH) for HCC with cirrhosis by collecting high-quality case-matched studies.

## Methods

### Search Strategy

This systematic review was conducted in accordance with the Preferred Reporting Items for Systematic Reviews and Meta-Analyses (PRISMA) guidelines (19). Electronic databases including PubMed, Embase, and Cochrane Library were searched. The search strategy for Pubmed was as follows: (((((“Minimally Invasive Surgical Procedures”[Mesh]) OR “Laparoscopy”[Mesh])) AND “Liver Cirrhosis”[Mesh]) AND “Liver Neoplasms”[Mesh]) and similar strategy was performed in other databases. The references of the retrieved results were also manually reviewed to obtain more related articles as possible. The final search was conducted in Mar 2021. No institutional review board approval or patient written consent was necessary because only published data were used.

### Study Selection

Case-matched studies written in English and comparing the outcomes of OH *vs* LH for HCC in patients with cirrhosis were considered for inclusion. The exclusion criteria were as follows: (i). reviews, editorials, case reports, abstracts, or letters; (ii). studies including patients without cirrhosis or those with unproven cirrhosis; (iii). studies including patients who underwent robotic or hybrid procedures; (iv). overlapped studies; (v). studies that did not report at least three of the primary outcomes.

### Data Extraction

After the initial screening, full-text versions of the selected articles were obtained. Two reviewers (SX and KC), as well as an independent third reviewer (YP) in cases in which consensus could not be reached, individually assessed each article and rejected those that failed to meet the inclusion criteria. The following items were extracted: year of publication, study design, sample size, country of study, patient characteristics, and outcome measures. The primary outcomes were overall complication rate, major complication rate, postoperative mortality, overall survival (OS) rate, and disease-free survival (DFS) rate. The secondary outcomes were operation time, blood loss, transfusion rate, length of hospitalization (LOH), minor complication rate, postoperative ascites, and postoperative liver failure (POLF). The Newcastle-Ottawa scale (NOS) was used to evaluate the quality of observational studies (http://www.ohri.ca/programs/clinical_epidemiology/oxford.asp). The NOS scores were ≥7, were considered of high quality. According to previous studies, minor resection was defined as hepatectomy of fewer than three sections and major resection was defined as hepatectomy of more than three sections (20–22). Clavien–Dindo classification was used to grade postoperative complications and a major complication was defined as Clavien–Dindo ≥3; otherwise, the complication was defined as minor (23).

### Statistical Analysis

Dichotomous variables were evaluated using odds ratios (ORs) with 95% confidence intervals (95% CIs), and continuous variables were analyzed using the weighted mean differences (WMDs) with 95% CIs. The hazard ratio (HR) was used as a summary statistic for long-term outcomes (survival analysis), as described by Tierney et al. (24). Medians were converted to means using the formula described by Hozo et al. (25). According to the Higgins I^2^ statistic, heterogeneities <25, 25 to 50, and >50% were defined as low, moderate, and high, respectively (26). A fixed-effects model was used for studies with low or moderate statistical heterogeneity (27), whereas a random-effects model was used for studies with high statistical heterogeneity (28). Subgroup analysis was performed according to different resection types: major resection and minor resection. Funnel plots were used to estimate the potential publication bias. P<0.05 was considered statistically significant. The meta-analysis was performed using the STATA 12.0.

## Results

### Study Characteristics

**Figure 1:**
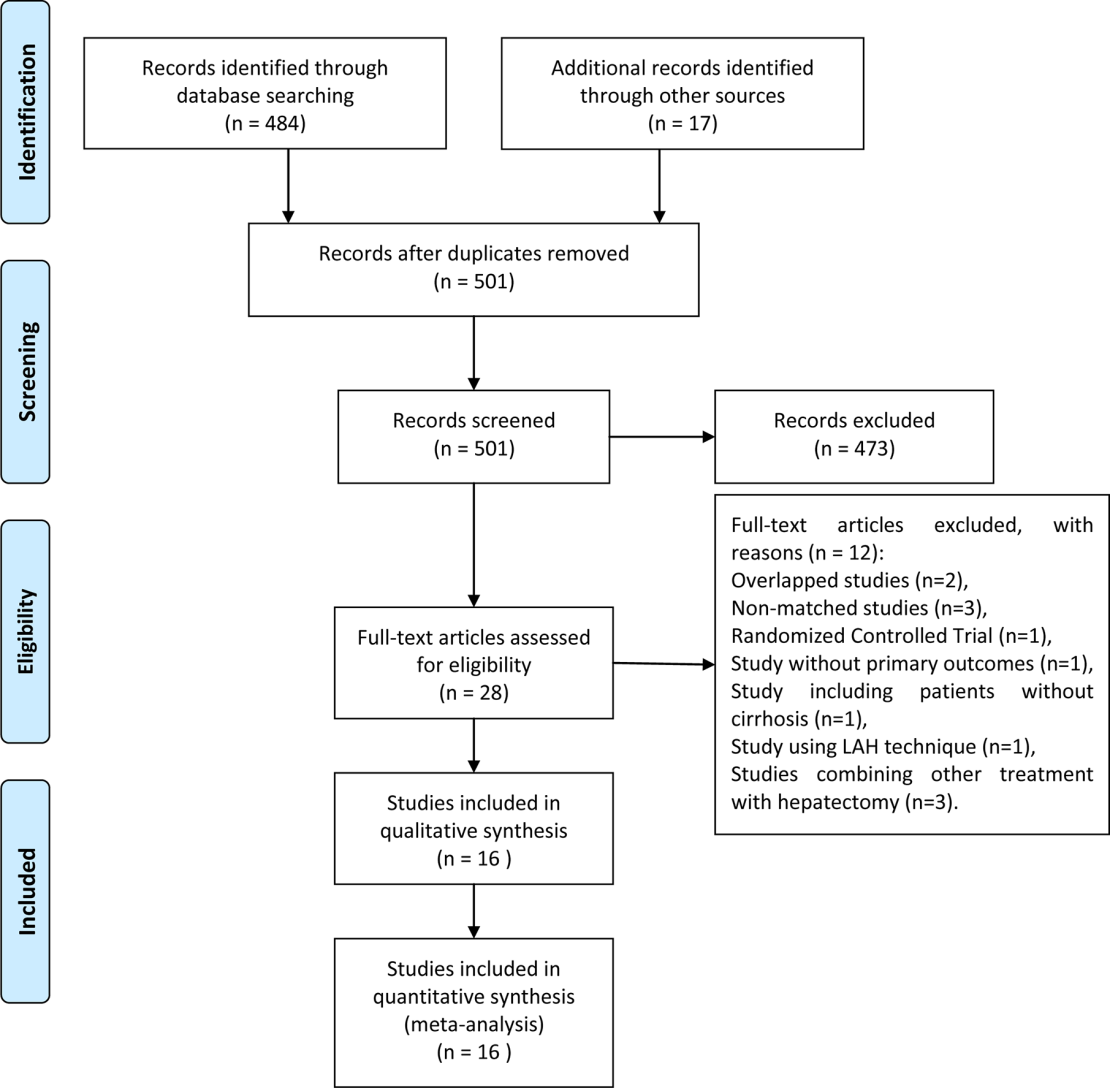
Flow chart of the studies included in the meta-analysis.

This meta-analysis was registered to PROSPERO (https://www.crd.york.ac.uk/prospero/) with an ID of CRD42020161775. The search strategy initially retrieved 501 records. After the exclusion of irrelevant studies by screening the abstracts, the full texts of 28 potentially relevant articles were obtained for assessment. Twelve studies were excluded due to overlapping data, inclusion of patients without cirrhosis, unavailable statistical data, non-comparative studies, non-case matched studies (8, 9, 29–38). Sixteen studies were eventually included (15–18, 39–50). The PRISMA flowchart of the literature review is presented in **Figure 1**.

The characteristics of the included studies are summarized in **Table 1**. A total of 1,975 patients from both Eastern and Western countries were pooled in this meta-analysis: 784 patients in the LH group and 1,191 patients in the OH group. To balance the basic characteristics, the propensity score matching method was used in 12 out of 16 retrospective studies, whereas the case-matched method was used in the others. Detail of matched characteristics was summarized in **Supplementary Table 1**. Nine studies focused on minor liver resection, and five studies reported outcomes limited to patients who underwent major liver resection. Ten studies reported the conversion rate of LH, which ranged from 0 to 34.21%. Surgical techniques including inflow occlusion method, parenchymal transection technique, and hemostasis method, were summarized in **Supplementary Table 2**. All studies were considered to be of adequate quality for the meta-analysis, as presented in **Table 2**.

**Table 1:** The basic characteristics of included studies.

study	year	Country	Study design	sample size (LH/OH)	Mean age (LH/OH)	Gender (M/F) (LH/OH)	Childs-Pugh A:B ratio (LH/OH)	tumor size (LH/OH)	tumor pattern (LH/OH)	conversion rate	resection type	Matched method
Belli et al.	2007	Italy	R	23 vs 23	59.5 vs 62.4	13/10 vs 14/9	23/0 vs 23/0	3.1 vs 3.2	NA	0	minor	M
Truant et al.	2011	France	R	36 vs 53	60.6 vs 63.3	31/5 vs 47/6	36/0 vs 53/0	2.9 vs 3.1	34/2 vs 44/9	NA	minor	M
Memeo et al.	2014	France	R	45 vs 45	62 vs 60	35/10 vs 37/8	44/1 vs 43/2	3.2 vs 3.7	NA	0	minor	M
Komatsu et al.	2016	Japan	R	38 vs 38	61.5 vs 61.7	34/4 vs 33/5	38/0 vs 38/0	4.75 vs 8.5	19/19 vs 22/16	34.21%	major	M
Cheung et al.	2016	China	R	110 vs 330	60 vs 61	80/30 vs 258/72	110/0 vs 330/0	2.6 vs 2.85	100/10 vs 292/38	5.5%	minor	PSM
Jiang et al.	2016	China	R	59 vs 59	51 vs 50	42/17 vs 38/21	59/0 vs 59/0	3 vs 3	59/0 vs 59/0	5.1%	minor	PSM
Yoon et al.	2017	Korea	R	33 vs 33	56.03 vs 57.33	23/10 vs 26/7	33/0 vs 33/0	3.31 vs 2.96	NA	NA	major	PSM
Xu et al.	2018	China	R	32 vs 32	53.5 vs 52	28/4 vs 28/4	32/0 vs 32/0	4 vs 6.2	29/3 vs 29/3	NA	major	PSM
Kim et al.	2018	Korea	R	18 vs 36	55.7 vs 54.6	13/5 vs 22/14	18/0 vs 36/0	2.9 vs 3.66	18/0 vs 36/0	0	minor	PSM
Sandro et al.	2018	Italy	R	75 vs 75	68.6 vs 67.1	33/42 vs 24/51	65/10 vs 63/12	2.5 vs 2.5	66/9 vs 65/10	7.6%	minor	PSM
Delvecchio et al.	2020		RM	38 vs 84	75 vs 74.3	29/9 vs 61/23	37/1 vs 82/2	4 vs 7	33/5 vs 68/18	NA	major	PSM
Cheung et al.	2020	China	R	24 vs 96	63 vs 62	20/4 vs 81/15	24/0 vs 96/0	4.5 vs 4.8	18/6 vs 75/21	NA	major	PSM
Hobeika et al.	2020	France	R	124 vs 124	63 vs 63	98/26 vs 101/13	NA	NA	NA	16.8%	minor	PSM
Yamamoto et al.	2020	Japan	R	58 vs 58	71 vs 72	39/19 vs 30/28	45/13 vs 45/13	1.7 vs 1.6	NA	NA	minor	PSM
Inoue et al.	2020	Japan	R	28 vs 28	73 vs 72	19/9 vs 18/10	28/0 vs 27/1	2.4 vs 2.4	NA	12.70%	NA	PSM
Fu et al.	2021	China	R	43 vs 77	52 vs 56	33/10 vs 59/18	43/0 vs 70/0	2.5 vs 2.5	NA	2.0%	NA	PSM

LH laparoscopic hepatectomy, OH open hepatectomy, M male, F female, NA not available, R retrospective, RM retrospective multicenter, PSM propensity score-matched.

**Table 2:** The qualities of included studies evaluated using the Newcastle-Ottawa Quality Assessment Scale.

Study	Selection	Comparability	Outcomes	Total
	1. Representativeness of exposed cohort2. Selection of nonexposed cohort3. Ascertainment of exposure4. Outcome not present at the start of the study		1. Assessment of outcomes2. Length of follow-up3. Adequacy of follow-up	
Belli et al.	****	**	**	********
Truant et al.	****	**	***	*********
Memeo et al.	****	**	***	*********
Cheung et al.	****	**	***	*********
Jiang et al.	****	**	*	*******
Komatsu et al.	****	**	**	********
Yoon et al.	****	**	**	********
Sandro et al.	****	**	**	********
Xu et al.	****	**	**	********
Kim et al.	****	**	**	********
Delvecchio et al.	****	**	***	*********
Cheung et al.	****	**	***	*********
Hobeika et al.	****	**	*	*******
Yamamoto et al.	****	**	***	*********
Inoue et al.	****	**	*	*******
Fu et al.	****	**	*	*******

*1 score.

### Intraoperative Outcomes

**Figure 2:**
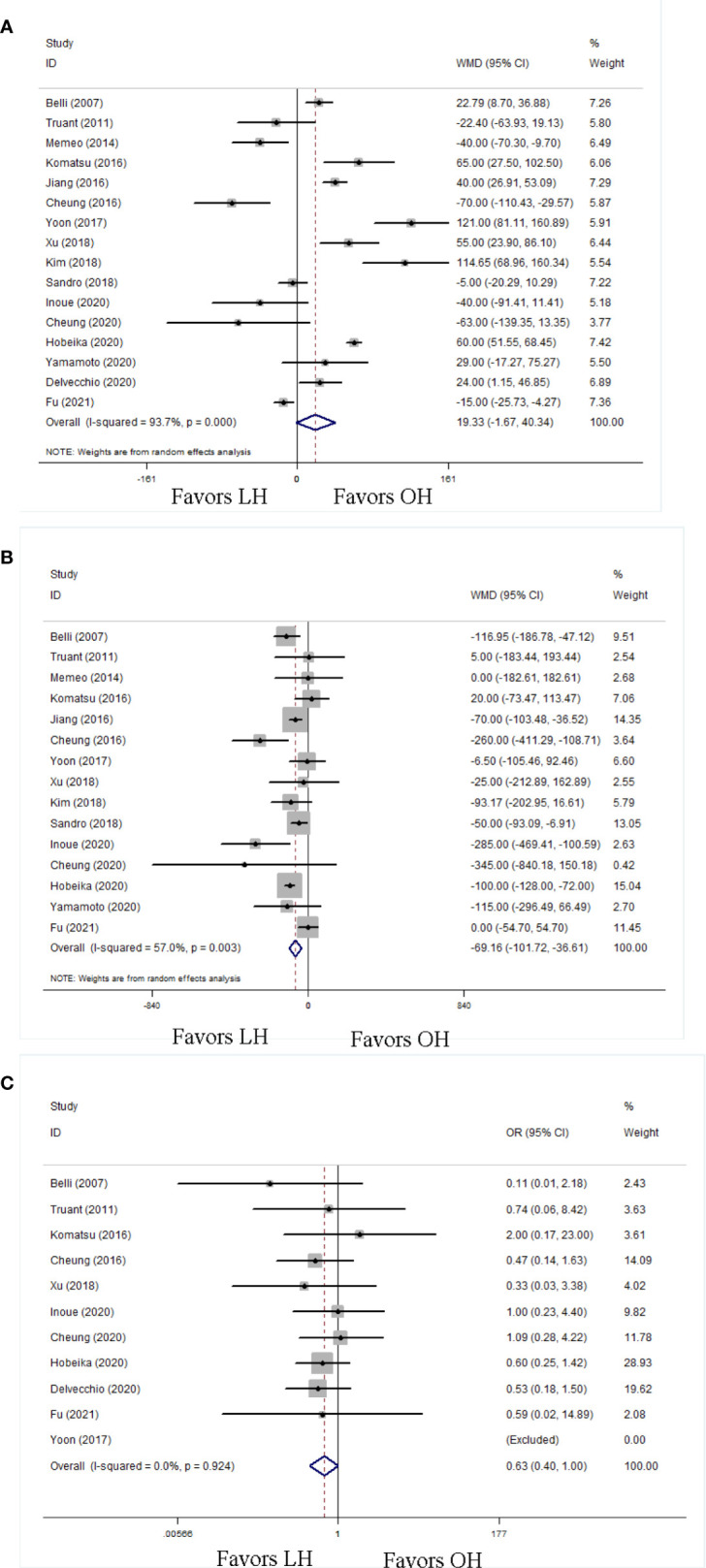
Forest plots of intraoperative outcomes, **(A)** operation time, **(B)** blood loss, **(C)** transfusion rate.

All 16 pooled studies reported the operation time. Compared with the OH group, the LH group achieved a comparable operation time (WMD 19.33, 95% CI −1.67 to 40.34; P = 0.07; **Figure 2A**). According to 15 studies reporting intraoperative blood loss, our meta-analysis found blood loss was less in the LH than that in the OH groups (WMD −69.16; 95% CI −101.72 to −36.61; P < 0.01; **Figure 2B**). Similarly the occurrence of transfusion in LH was less than that in OH (OR 0.63; 95% CI 0.40 to 1.00; P = 0.05; **Figure 2C**).

### Postoperative Outcomes

**Figure 3:**
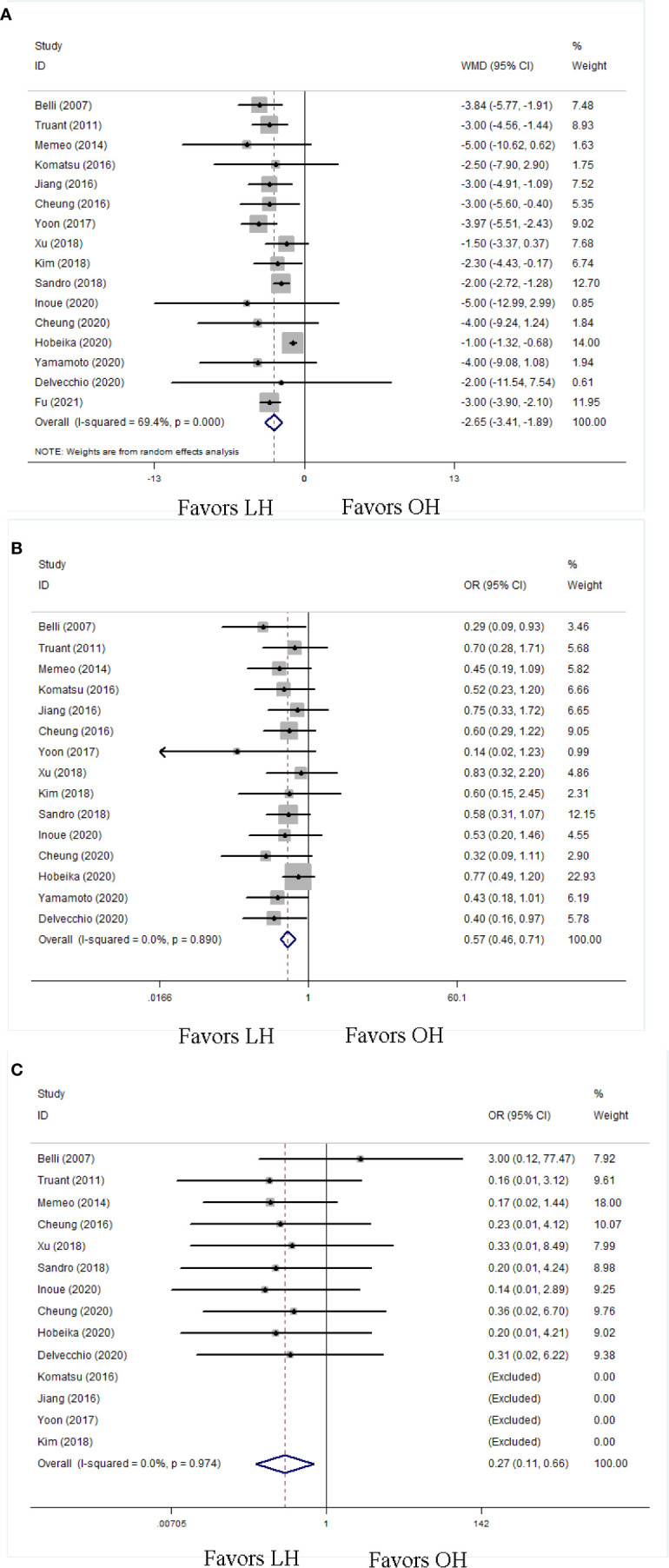
Forest plots of postoperative outcomes, **(A)** length of postoperative hospitalization, **(B)** overall postoperative complication, **(C)** postoperative mortality.

A shorter LOH was observed in LH (WMD −2.65; 95% CI −3.41 to −1.89; P < 0.01; **Figure 3A**). Postoperative complications were recorded in fifteen studies. The LH group had a decreased risk of overall postoperative complications (OR 0.57; 95% CI 0.46 to 0.71; P < 0.01; **Figure 3B**). Moreover, 15 studies reported postoperative mortalities. On the basis of these data, LH had a lower mortality rate (OR 0.27; 95% CI 0.11 to 0.66; P < 0.01; **Figure 3C**). To clarify the influence of LH on postoperative complications, we classified postoperative complications into minor complications and major complications.

**Figure 4:**
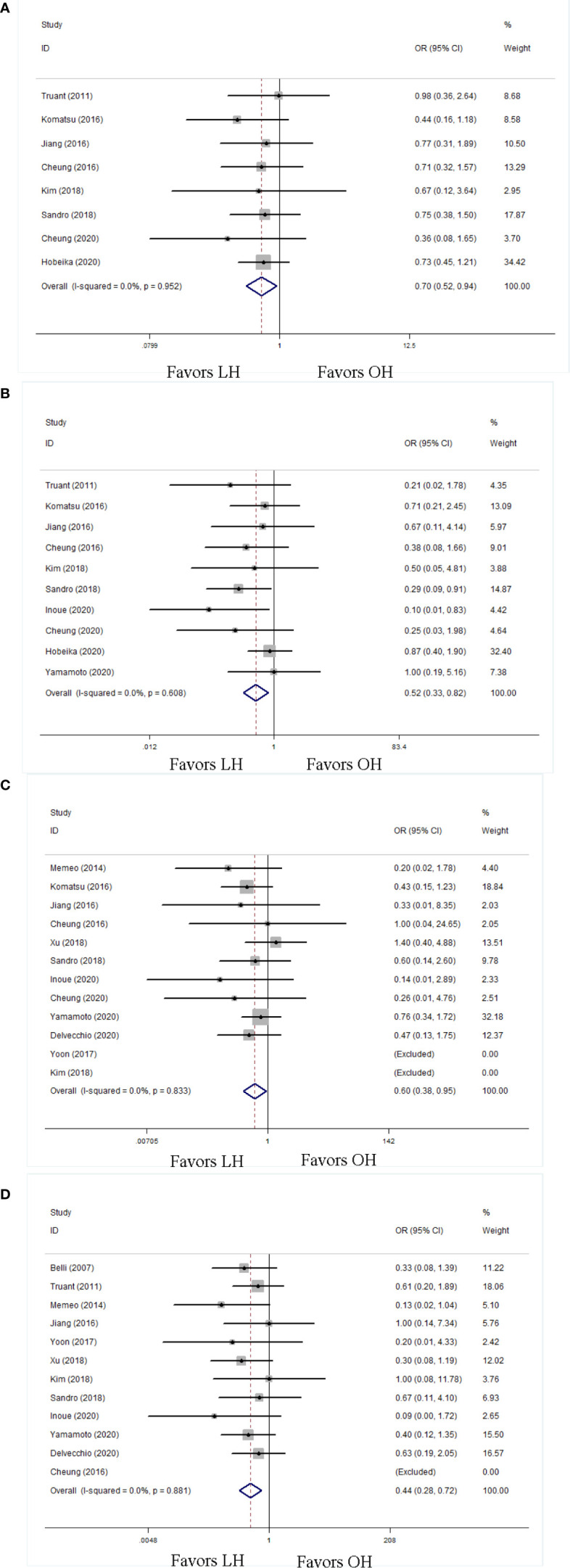
Forest plots of postoperative complication in detail, **(A)** minor complication, **(B)** major complication, **(C)** postoperative liver failure, **(D)** ascites.

With respect to the overall postoperative complications, the LH group had more favorable minor complication rate (OR 0.70; 95% CI 0.53 to 0.94; P = 0.02; **Figure 4A**) and major complication rate (OR 0.52; 95% CI 0.33 to 0.82; P < 0.01; **Figure 4B**) than OH. We also evaluated some detailed complications specifically associated with liver resection in patients with cirrhosis, including POLF and ascites. The LH group had less POLF (OR 0.60; 95% CI 0.38 to 0.95; P = 0.03; **Figure 4C**) and ascites (OR 0.44; 95% CI 0.28 to 0.72; P < 0.01; **Figure 4D**) than the OH group.

### Long-Term Outcomes

**Figure 5:**
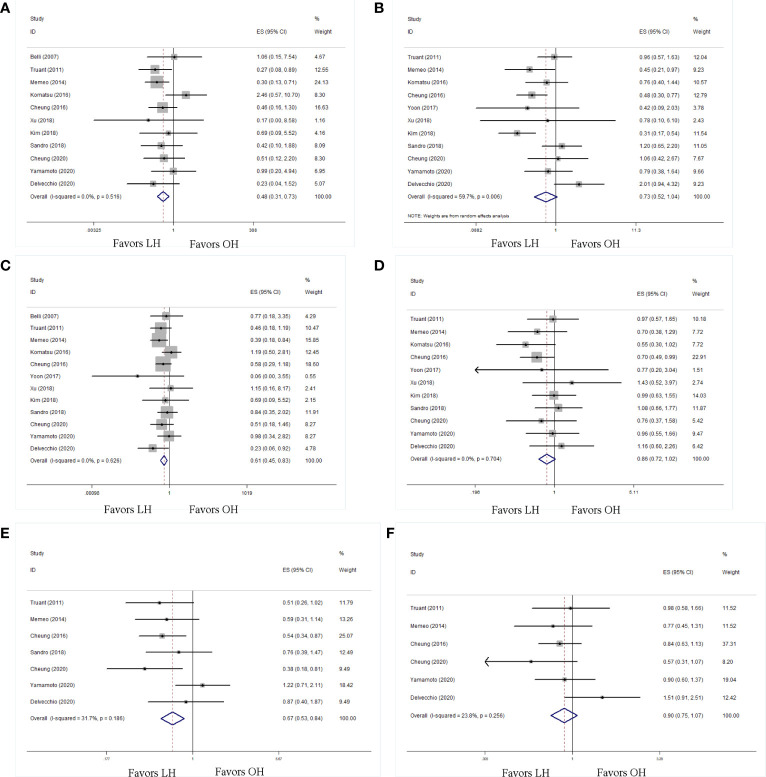
Forest plots of long-term outcomes, **(A)** 1-y overall survival rate, **(B)** 1-y disease-free survival rate, **(C)** 2-y overall survival rate, **(D)** 2-y disease-free survival rate, **(E)** 5-y overall survival rate, **(F)** 5-y disease-free survival rate.

Twelve studies reported the long-term outcomes including OS and DFS rates. The data showed that LH had more favorable 1-, 2-, and 5-year OS rate (1-year: HR 0.48; 95% CI 0.31 to 0.73; P < 0.01; **Figure 5A**; 2-year: HR 0.61; 95% CI 0.45 to 0.83; P < 0.01; **Figure 5C**; 5-year: HR 0.67; 95% CI 0.53 to 0.85; P < 0.01; **Figure 5E**) than OH. As for the DFS rate, LH had comparable outcomes to OH in terms of 1-, 2-, and 5-year DFS rates (1-year: HR 0.73; 95% CI 0.52 to 1.04; P = 0.08; **Figure 5B**; 2-year: HR 0.86; 95% CI 0.73 to 1.02; P =0.08; **Figure 5D**; 5-year: HR 0.90; 95% CI 0.75 to 1.07; P = 0.23; **Figure 5F**).

### Subgroup Analysis

Given that the included studies enrolled patients who underwent different extents of liver resection, subgroup analysis was conducted according to the resection extent (minor or major resection), as shown in **Table 3**. In accordance with the overall analysis, LH was associated with less blood loss, shorter LOH, fewer postoperative complications and mortalities, better 1-year, 2-year, and 5-year OS rate in minor resection subgroup analysis. Notably, in the major resection subgroup analysis, the LH group had a longer operation time, shorter LOH, and fewer postoperative complications than the OH group. Moreover, there was no difference in the OS and DFS rates between the LH and OH groups in the major resection subgroup analysis.

**Table 3:** Subgroup analysis of outcomes based on the surgical extents.

Outcomes	Included studies	Sample size	I2	Pooled mode	Pooled effect	P value
Operation time						
All	16	1975	93.7%	Random	WMD:19.33(-1.67,40.34)	0.07
Minor resection	9	1351	93.9%	Random	WMD:14.80(-11.24,40.85)	0.27
Major resection	5	448	84.7%	Random	WMD:47.24(5.52,89.00)	0.03
Blood loss						
All	15	1853	57.0%	Random	WMD:-69.16(-101.72,-36.61)	<0.01
Minor resection	9	1351	34.3%	Fixed	WMD:-84.75(-112.22,-57.29)	<0.01
Major resection	4	326	0.0%	Fixed	WMD:-1.97(-65.34,61.40)	0.95
Transfusion						
All	10	1381	7.3%	Fixed	OR:0.63(0.40,1.00)	0.05
Minor resection	4	823	0.0%	Fixed	OR:0.52(0.27.1.02)	0.06
Major resection	4	382	0.0%	Fixed	OR:0.71(0.34,1.49)	0.36
LOH						
All	16	1975	69.4%	Random	WMD:-2.65(-3.41,-1.89)	<0.01
Minor resection	9	1351	70.8%	Random	WMD:-2.45(-3.33,-1.57)	<0.01
Major resection	5	448	4.6%	Random	WMD:-2.99(-4.11,-1.86)	<0.01
Overall complication						
All	15	1859	0.0%	Fixed	OR:0.57(0.46,0.71)	<0.01
Minor resection	9	1351	0.0%	Fixed	OR:0.61(0.48,0.78)	<0.01
Major resection	5	448	0.0%	Fixed	OR:0.47(0.30,0.75)	<0.01
Minor complication						
All	8	1295	0.0%	Fixed	OR:0.70(0.53,0.94)	0.02
Minor resection	6	1099	0.0%	Fixed	OR:0.76(0.55,1.03)	0.08
Major resection	2	196	0.0%	Fixed	OR:0.41(0.18,0.95)	0.04
Major complication						
All	10	1467	0.0%	Fixed	OR:0.52(0.33,0.82)	<0.01
Minor resection	7	1215	0.0%	Fixed	OR:0.57(0.34,0.94)	0.03
Major resection	2	196	0.0%	Fixed	OR:0.54(0.19,1.56)	0.26
Mortality						
All	10	1425	0.0%	Fixed	OR:0.27(0.11,0.66)	<0.01
Minor resection	6	1063	0.0%	Fixed	OR:0.26(0.08,0.83)	0.02
Major resection	3	306	0.0%	Fixed	OR:0.34(0.06,1.94)	0.22
POLF						
All	10	1352	0.0%	Fixed	OR:0.60(0.38,0.95)	0.03
Minor resection	5	914	0.0%	Fixed	OR:0.63(0.33,1.21)	0.17
Major resection	4	382	0.0%	Fixed	OR:0.60(0.31,1.17)	0.14
Ascites						
All	11	971	0.00%	Fixed	OR:0.44(0.28,0.72)	<0.01
Minor resection	7	663	0.00%	Fixed	OR:0.48(0.27,0.86)	0.01
Major resection	3	252	0.00%	Fixed	OR:0.43(0.18,1.02)	0.05
1-year OS						
All	11	1367	32.90%	Fixed	HR:0.48(0.31,0.73)	<0.01
Minor resection	7	985	0%	Fixed	HR:0.42(0.26,0.68)	<0.01
Major resection	4	382	36.4%	Fixed	HR:0.72(0.30,1.74)	0.46
2-year OS						
All	12	1433	0.00%	Fixed	HR:0.61(0.45,0.83)	<0.01
Minor resection	7	985	0%	Fixed	HR:0.59(0.42,0.85)	<0.01
Major resection	5	448	31.7%	Fixed	HR:0.66(0.37,1.17)	0.16
5-year OS						
All	7	1127	31.70%	Fixed	HR:0.67(0.53,0.85)	<0.01
Minor resection	5	885	35%	Fixed	HR:0.69(0.53,0.90)	<0.01
Major resection	2	242	55.8%	Random	HR:0.57(0.26,1.30)	0.18
1-year DFS						
All	11	1387	59.70%	Random	HR:0.73(0.52,1.04)	0.08
Minor resection	6	939	67.10%	Random	HR:0.63(0.41,0.96)	0.03
Major resection	5	448	22.3%	Fixed	HR:1.03(0.69,1.56)	0.88
2-year DFS						
All	11	1387	0%	Fixed	HR:0.86(0.73,1.02)	0.08
Minor resection	6	939	0%	Fixed	HR:0.87(0.72,1.05)	0.15
Major resection	5	448	0%	Fixed	HR:0.83(0.59,1.17)	0.29
5-year DFS						
All	6	781	23.80%	Fixed	HR:0.90(0.75,1.07)	0.23
Minor resection	4	735	0%	Fixed	HR:0.87(0.71,1.06)	0.16
Major resection	2	242	81.9%	Random	HR:0.95(0.37,2.44)	0.91

LOH length of hospitalization, CI confidence interval, WMD weighted mean difference, OR odds ratio, POLF postoperative liver failure, HR hazard ratio, OS overall survival, DFS disease-free survival disease-free survival.

### Sensitivity Analysis and Publication Bias

**Figure 6:**
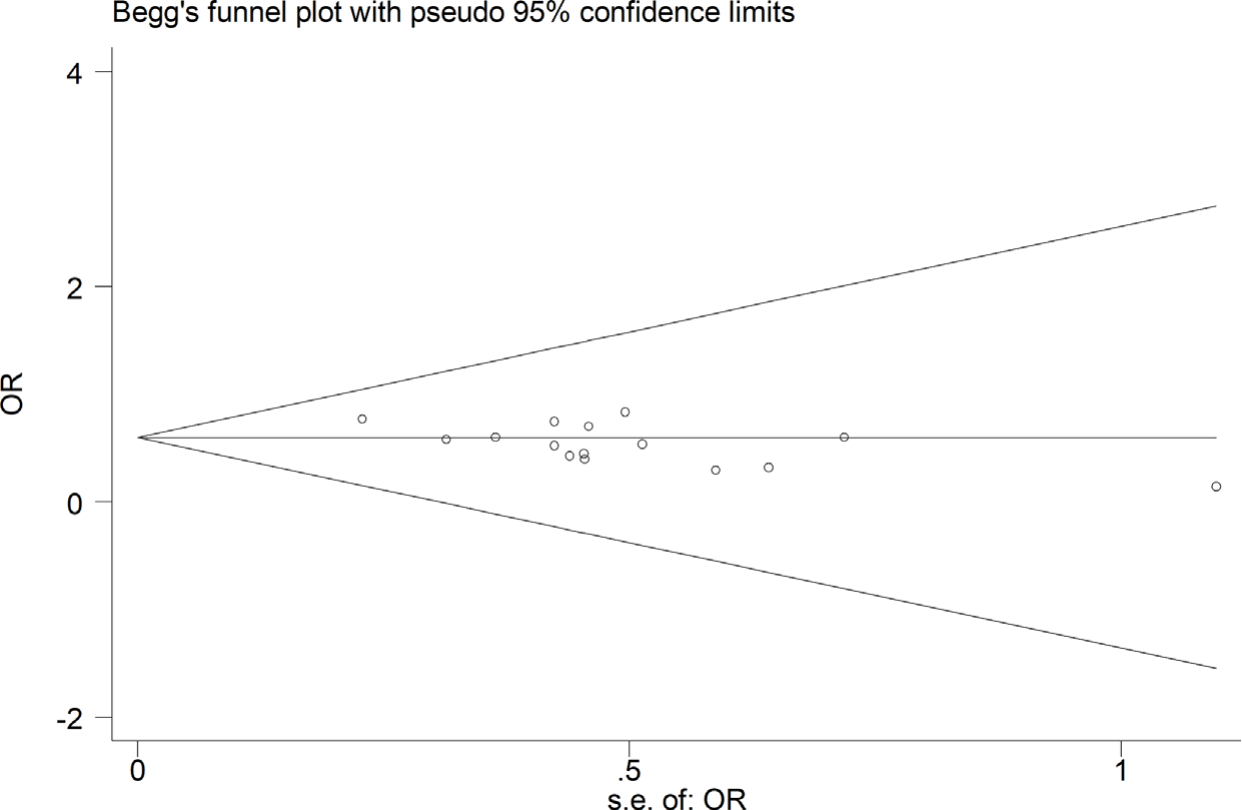
Funnel plots of postoperative complication.

Sensitivity analyses were conducted by excluding the highest-weighted study in each pooled analysis. These exclusions did not alter the results of cumulative analyses. A funnel plot based on overall postoperative complications was performed to assess publication bias. No significant publication bias was detected by visual inspection of the funnel plot, in which the pooled studies were almost symmetrical and none of them were outside the 95% CI (**Figure 6**).

## Discussion

OH is a well-established curative treatment for HCC. However, patients with poor liver functional reserve, such as those with cirrhosis, are at higher risk of undergoing OH with a large surgical incision, wide extent of resection, and relatively large amount of blood loss. LH is emerging as a promising alternative approach for HCC patients with cirrhosis. Several previous meta-analyses have evaluated the advantages and disadvantages of LH (**Table 4**). Studies by Twaij et al. and Chen et al. identified significantly decreased overall postoperative complications, mortality, blood loss, and LOH in the LH group (12, 13). Goh et al. reported that LH was associated with better oncological outcomes (51). However, most studies included in those meta-analyses were retrospective studies with small sample sizes, which are prone to biases. Recently, several high-quality articles comparing LH and OH for HCC with cirrhosis have been published (14–18). To minimize the selection bias, this systematic review and meta-analysis pooled 16 case-matched retrospective studies. Comparisons were made between LH and OH for HCC in patients with cirrhosis, along with subgroup analysis according to different surgical extents.

**Table 4:** Summary of outcomes reported by previous meta-analysis and present meta-analysis.

Study	Latest literature search	Included studies	Study characteristics	Operation time	Blood loss	Blood transfusion	postoperative morbidity	postoperative mortality	LOH	1-year OS	5-year OS	1-year DFS	5-year DFS
Twaij et al.	2013.8	4	R&RM	E	FLH	FLH	FLH	NA	FLH	NA	NA	NA	NA
Chen et al.	2015.3	7	R&RM	E	FLH	FLH	FLH	E	FLH	E	FLH	E	E
Goh et al.	2016.11	5	R&RM	NA	NA	NA	NA	NA	NA	FLH	FLH	FLH	E
Present study	2021.3	16	RM	E	FLH	FLH	FLH	FLH	FLH	FLH	FLH	E	E

LOH length of hospitalization, OS overall survival, DFS disease-free survival, R retrospective study, RM retrospective matched study, RCT randomized clinical trial, E equivalent, FLH favors laparoscopic hepatectomy, NA not available.

Consistent with previous studies, the main findings obtained from our meta-analysis showed that patients who underwent LH presented notable oncological advantages in terms of 1-, 2-, and 5-year OS and 1-year DFS. In addition, our meta-analysis showed that LH was associated with lower postoperative morbidity, lower mortality, less blood loss, and shorter LOH than OH.

The primary concern with LH was bleeding control during transection in the setting of cirrhosis. The impact of cirrhosis on portal vein pressure and coagulation, and the movement restriction in laparoscopic surgery, make bleeding control challenging and increase the conversion risk. Truant et al. reported that uncontrolled bleeding accounted for 57.1% (4/7) of total conversion (42). Similarly, Sandro et al. also reported that one-third (2/7) of patients underwent conversion because of bleeding (15). With the accumulation of surgical experience, bleeding control during transection has been established by using the Pringle maneuver, compression with or without hemostatic material, clipping, suturing, temporary clamp for vessels, and various energy devices. Simultaneously, decreased intraoperative blood loss has been achieved with the application of appropriate pneumoperitoneum pressure, which reduces venous bleeding, and a magnified operating view, which allows meticulous manipulation. In this meta-analysis, the blood loss in the LH group was less than that in the OH group, as reported in previous studies. The considerable decrease in blood loss with the LH procedure means a decreased risk of transfusion. Accordingly, a lower transfusion rate in the LH group was observed in the present study.

Decreased blood loss, avoidance of large incisions and meticulous manipulation alleviate the surgical trauma. The minimally invasive approach reduces the risk of acute or delayed systematic adverse events and subsequent postoperative morbidity and mortality. The overall complication rate of LH was approximately 22.8% (169/741), which was significantly lower than that of OH (34.9%, 389/1,114). Recently, Goh et al. examined 400 cases of LH and reported a postoperative morbidity of 18.8%, which is equivalent to the present study (52). A similar result was observed in that OH had a nearly four-fold risk of postoperative death in comparison with LH (OR = 0.28).

Hepatectomy can lead to refractory ascites in patients with cirrhosis, which can be fatal. By preserving the integrity of the abdominal wall and reducing surgery-induced injury to the area surrounding the liver, disruption of collateral blood and lymphatic flow is minimized in the laparoscopic approach. Further analysis of postoperative complications revealed that the LH group had less postoperative ascites. The LH group had fewer major and minor complications as than the OH group. Furthermore, in the setting of LH, minor complications were predominant, accounting for 75.8% (91/120) of the overall complications, which was significantly higher than that in OH (68.2%, 176/258). Therefore, it can be deduced that LH is technically safe and tends to have fewer and milder complications.

Reduced surgical trauma, fewer postoperative events, and enhanced recovery resulted in shorter LOH and lower medical costs. More importantly, the present study demonstrated that patients undergoing LH had better oncological outcomes, including 1-, 2-, 5-year OS and 1-year DFS. Although no statistical difference was found in 2- and 5-year DFS owing to the inclusion of limited studies, a trend of favoring LH was observed. We speculated that the better prognosis of LH patients might lie in the less compression during laparoscopic manipulation, which prevented tumor cell metastasis. In addition, the minimally invasive approach resulted in faster recovery of the immune and nutritional status, which may also contribute to better prognosis.

Unlike previous meta-analyses on this issue, the present study performed subgroup analysis based on the surgical extent, which was necessary to eliminate such heterogeneity among the studies. The present study found that the results of subgroup analysis based on minor resection were in line with the results of the overall analysis, however, the results of subgroup analysis based on major resection should be cautiously interpreted, although only three studies were included. As expected, LMH was a potential alternative to its open counterpart, and it maintained the advantage of shorter LOH and fewer postoperative complications as in laparoscopic minor hepatectomy. However, LMH had a longer operation time than the open approach, suggesting that this procedure is technically demanding. Notably, Komatsu et al. reported a conversion rate of 34.21% in the LMH group, reflecting the steep learning curve of LMH in the setting of HCC with cirrhosis. Comprehensive liver function assessment and a good understanding of the liver anatomy, as well as ample surgical expertise, are the most important factors for successful LMH. Emerging evidence proving the value of LMH may lead to the expansion of the indication of LH to HCC patients with cirrhosis.

Our review has notable strengths as follows: (i) all included studies were case-matched studies, which balanced the baseline characteristics and reduced the selection bias, (ii) more detailed data than in other meta-analyses were extracted and analyzed, and (iii) “HR” instead of “OR” was applied in analyzing time-to-event data, such OS and DFS. Nevertheless, the present meta-analysis also had several limitations. First, most of the included studies were retrospective studies which adversely affected the overall quality of the evidence. Although the baseline characteristics of confounding factors were balanced in all included retrospective studies, the allocation of patients was rarely described in the included studies, which inevitably resulted in selection bias. Second, none of the included studies prospectively has calculated the sufficient sample size to identify differences between OH and LH. Several studies with small sample sizes presented the initial experience of surgeons in performing LH, although those surgeons might have a high level of expertise in OH. The lack of sufficient sample size and quality control of the surgical techniques might also bring bias. Third, LH is considered as an emerging and potentially better alternative to OH. It can’t be guaranteed that all results, including LH with poor outcomes, were reported, and no mandatory registration is required in observational studies, which can be a source of publication bias.

## Conclusion

In summary, this systematic review and meta-analysis comparing LH and OH demonstrated that LH can be safely performed in selected HCC patients with cirrhosis. LH offers favorable short-term outcomes and long-term oncological outcomes in minor liver resections. Although LMH seems to offer some advantages over the open approach, concerns about surgical and oncological safety remain. More evidence on LMH is warranted before expanding its indication to patients with cirrhosis.

## Data Availability Statement

The raw data supporting the conclusions of this article will be made available by the authors, without undue reservation.

## Ethics Statement

This research was an analysis of published data and did not require informed consent. Ethics approval and consent to participate were not applicable in this research.

## Author Contributions

Conception and design: X-JC and YP. Administrative support: X-JC. Provision of study material or patients: YP, KC, and S-JX. Collection and assembly of data: KC, S-JX, and J-QC. Data analysis and interpretation: YP. Manuscript writing: all authors. All authors contributed to the article and approved the submitted version.

## Funding

This work was supported by the National Natural Science Foundation of China under Grant No. 81701911.

## Conflict of Interest

The authors declare that the research was conducted in the absence of any commercial or financial relationships that could be construed as a potential conflict of interest.
