# Supplementary material for: Efficacy of Laparoscopic Hepatectomy versus Open Surgery for Hepatocellular Carcinoma With Cirrhosis: A Meta-analysis of Case-Matched Studies
Source: Front Oncol. 2021 May 7;11:652272. doi: 10.3389/fonc.2021.652272 (PMC8139628; doi:10.3389/fonc.2021.652272)
Supplement: Supplementary file 1 [file DataSheet_1.pdf]

Supplementary Table 1 Matched characteristics of included studies

Study	Matched characteristics
Belli et al.	age, sex, ASA score, liver function, tumor size, tumor location and type of resection
Truant et al.	tumor size, tumor number and type of resection
Memeo et al.	liver function, underlying liver disease, AFP, tumor size, tumor number, type of resection and pathological characteristics
Komatsu et al.	age, sex, BMI, ASA score, liver function, tumor size, tumor number and operative procedure
Jiang et al.	age, sex, BMI, ASA score, underlying liver disease, liver function, tumor location and type of resection.
Cheung et al.	NA
Xu et al.	age, sex, BMI, ASA score, comorbidities, liver function, previous abdominal surgery history, tumor size and tumor location
Kim et al.	age, BMI, liver function, tumor size, tumor location, tumor number and presence of microvascular invasion
Sandro et al.	age, ECOG performance status, liver function, AFP, tumor size, tumor number and type of resection
Delvecchio et al.	sex, ASA score, comorbidities, liver function, tumor size and tumor number
Fu et al.	age, liver function, tumor size, tumor location, tumor capsule, tumor diferentiation, and resection type
Inoue et al.	age, sex, BMI, ASA score, underlying liver disease, liver function, tumor size, tumour location and tumor number
Cheung et al.	age, sex, comorbidities, underlying liver disease, liver function, tumor size, tumor number and tumor stage
Hobeika et al.	ASA score, liver function, type of resection and difficulty grade
Yamamoto et al.	tumor size, tumor number and difficulty grade
Yoon et al.	age, sex, ASA score, comorbidities, liver function, AFP and history of upper abdominal surgery

ASA American Society of Anesthesiologists, BMI body mass index, ECOG Eastern Cooperative Oncology Group, AFP a-fetoprotein, NA not available.

Supplementary Table 2 Surgical techniques of included studies

Study	Inflow occlusion method		Parenchymal transection technique		Hemostasis method	
	L	O	L	O	L	O
Belli et al.	PM when needed	PM when needed	Harmonic scalpel or a Ligasure device	Crushing forceps	Bipolar electrocoagulation	Bipolar electrocoagulation
Truant et al.	PM	PM	Harmonic scalpel	Harmonic scalpel or crushing forceps	Bipolar electrocoagulation	Bipolar electrocoagulation
Memeo et al.	PM when needed	PM	harmonic scalpel and bipolar forceps	Ultrasonic dissector		Bipolar electrocoagulation
Komatsu et al.	PM when needed	PM when needed	Ultrasonic dissector	Ultrasonic dissector	Bipolar electrocoagulation	Bipolar electrocoagulation
Jiang et al.	NA	NA	NA	NA	NA	NA
Cheung et al.	Individual isolation of the liver inflow	NA	Ultrasonic dissector and CUSA	CUSA	Metal clips, diathermy and sutures	Electrocautery, argon beam, or sutures
Xu et al.	PM	PM	Harmonic scalpel,CUSA or LigaSure	CUSA or clamp crushing	Clips	Bipolar electrocoagulation
Kim et al.	PM	PM	CUSA and other energy device	CUSA and other energy device	Clips	Monopolar electrocoagulation, argon beam, or nonabsorbable sutures

Sandro et al.	NA	NA	Harmonic scalpel,CUSA or LigaSure	NA	NA	clips and monofilamentstiches
Delvecchio et al.	PM	PM	Electrocoagulation, ultrasound, radiofrequency or combined energy	Electrocoagulation, ultrasound, radiofrequency or combined energy		
Fu et al.	PM	PM when needed	harmonic scalpel	harmonic scalpel	clips,bipolar electrocoagulation and sutures	Electrocoagulation or sutures
Inoue et al.	PM when needed	NA	Ultrasonic dissector	NA	Electrocautery, clips, or sutures.	NA
Cheung et al.	PM when needed	PM when needed	CUSA and other energy devices	CUSA and other energy devices	Electrocautery, argon beam or suturing	Electrocautery, argon beam or suturing
Hobeika et al.	NA	NA	NA	NA	NA	NA
Yamamoto et al.	PM	PM	Ultrasonic surgical aspirator and bipolar forceps	Ultrasonic surgical aspirator and bipolar forceps	NA	NA
Yoon et al.	PM when needed	PM when needed	Harmonic Scalpel, CUSA and other energy device	CUSA		Monopolar electrocoagulation, argon beam, clips, or suturing.

CUSA Cavitron ultrasonic surgical aspirator, NA not available, PM Pringle maneuver.
